# Mutation analysis of *NANOS3* in Brazilian women with primary ovarian failure

**DOI:** 10.6061/clinics/2016(12)03

**Published:** 2016-12

**Authors:** Braian Lucas A Sousa, Mirian Yumie Nishi, Mariza Gerdulo Santos, Vinicius Nahime Brito, Sorahia Domenice, Berenice B Mendonca

**Affiliations:** Hospital das Clínicas da Faculdade de Medicina da Universidade de São Paulo, Disciplina de Endocrinologia, Unidade de Endocrinologia do Desenvolvimento, Laboratório de Hormônios e Genética Molecular LIM/42, São Paulo/SP, Brazil

**Keywords:** Primary Amenorrhea, *NANOS3*, Gonadal Dysgenesis, Primary Ovarian Failure, Sexual Differentiation

## Abstract

**OBJECTIVES:**

Primary ovarian failure is a rare disorder, and approximately 90% of cases are of unknown etiology. The aim of this study was to search for mutations in *NANOS3,* a gene that was recently related to the etiology of primary ovarian failure, in a group of Brazilian women.

**METHODS:**

We screened for *NANOS3* DNA variants in 30 consecutive women who were previously diagnosed with primary ovarian failure, of unknown etiology and compared the results with those from 185 women with normal fertility. The *NANOS3* gene was amplified by polymerase chain reaction using pairs of specific primers and then sequenced. The resulting sequences were compared with control sequences available in the National Center for Biotechnology and Information database.

**RESULTS:**

No mutations in *NANOS3* were found in primary ovarian failure patients, but four previously described polymorphisms were identified at a similar frequency in the control and primary ovarian failure groups.

**CONCLUSIONS:**

Mutations in *NANOS3* were not associated with primary ovarian failure in the present cohort.

## INTRODUCTION

Primary ovarian failure (POF), also known as premature ovarian failure, premature ovarian insufficiency, premature menopause, or hypergonadotropic hypogonadism, is defined as the loss of function of the ovaries before age 40 1. POF is phenotypically characterized by the triad of primary or secondary amenorrhea, hypergonadotropic hypogonadism and hypoestrogenism.

There are several causes of POF, and the genetic causes are associated with a defect in ovarian development.

Sex determination in mammals is a dynamic and complex process that requires the interaction of multiple factors, and both primordial germ cells (PGCs) and somatic cells acquire sex-specific characteristics.

PGCs are essential for the differentiation and maintenance of the fetal ovary; when few germ cells enter the genital ridge during embryogenesis, the supporting lineage cells do not differentiate, generating strike ovaries 2-4. Because the differentiation of ovarian somatic cells relies on PGCs, genes involved in the differentiation and subsequent maintenance of PGCs are potential candidates for the etiology of 46,XX gonadal development disorders 5.

*Nanos homolog 3* was identified in PGCs of *Drosophila*, and the NANOS3 protein has anti-apoptotic activity during the migration of PGCs toward the gonad 6. Male and female mice that do not express *Nanos3* experience a complete loss of PGCs during gonadal development 7. The human *NANOS3* gene is located on the short arm of chromosome 19 (region 13.13) and has two exons.

A preliminary study in a Brazilian population identified a *NANOS3* c.385G>A homozygous mutation in two sisters with POF. *In vitro* analysis showed that the NANOS3 protein in these two patients had an impaired ability to prevent the apoptosis of PGCs, suggesting a possible etiology for the disorder 8.

Our objective was to search for inactivating *NANOS3* mutations in an additional group of 30 women with POF without apparent cause.

## MATERIALS AND METHODS

This was a cohort study performed in accordance with the ethical standards of the Ethics Committee of Hospital das Clínicas, University of São Paulo School of Medicine, Brazil (Protocol number 1226/07).

POF was defined as the loss of function of the ovaries before age 40 1. We invited 30 consecutive patients with the diagnosis of POF of unknown etiology who were followed in the Developmental Endocrinology Unit of Hospital das Clínicas, São Paulo, Brazil, to undergo screening for *NANOS3* mutations. Thyroid, adrenal and ovarian autoimmune disorders, as well as mutations in *FSHR*, *NR5A1*, *BMP15*, *GDF9*, *GATA4*, *DMRT1*, *FOXL2*, *LHCGR*, and *STRA8* and premutation of *FMR1*, had been ruled out in all these patients. All the patients had a normal 46,XX karyotype obtained by analysis of 50 metaphases.

Hormonal tests required for the POF diagnosis were performed at the Laboratory of Hormones and Molecular Genetics, LIM/42 of the HCFMUSP, using immunofluorometric assays (Delfia, Turku, Finland).

Basal gonadotropin levels were elevated in all patients, with predominantly high serum FSH levels ranging from 32 to 111 U/L and serum LH levels ranging from 13 to 107 U/L. Serum estradiol levels were low or within the follicular phase range in all patients.

The control group was composed of 185 women aged 18 to 65 years, all of whom had normal menstrual cycles and no history of menopause before 40 years of age or treatment for subfertility. The control group was screened for the *NANOS3* allelic variants found in the POF group.

Genomic DNA was extracted from peripheral blood leucocytes using the salting out procedure 9. The DNA concentration and purity were determined using the NanoPhotometer (Implen GmbH, Schatzbogen, Germany), considering an ideal 260/280 nm ratio of 1.75, and DNA integrity was assessed by 1% agarose gel electrophoresis, staining with SYBR Safe (Invitrogen Life Technologies Corporation, CA, USA) and visualization with ultraviolet light. *NANOS3* was amplified by PCR using specific pairs of primers located in the intronic regions of the gene (Table 1), and the PCR products were subjected to direct automated sequencing.

We performed PCR amplifications in a total volume of 25 µl (Table 2) as follows:

Exon 1: (94°C - 5 min) x 1 + (94°C - 30 sec; 58°C - 30 sec; 72°C - 45 sec) x 35 + (72°C - 10 min) x 1.

Exon 2: (94°C - 5 min) x 1 + (94°C - 30 sec; 57°C - 30 sec; 72°C - 45 sec) x 35 + (72°C - 10 min) x 1.

The PCR products were analyzed by electrophoresis on 1% agarose gels stained with SYBR Safe (Invitrogen Life Technologies Corporation, CA, USA) and visualized with ultraviolet light. To assess the DNA concentration of the PCR products, the intensity of the signal emitted by the bands was compared with the Low DNA Mass molecular weight marker (Invitrogen, Life Technologies Corporation, CA, USA). After amplification, PCR products were purified by incubation with ExoSAP-IT (GE Healthcare Life Sciences, Buckinghamshire, United Kingdom) for 15 min at 37°C and then 15 min at 80°C. DNA sequencing was performed according the protocol of the *ABI Prism BigDye Terminator Cycle Sequencing Ready Reaction Kit* (Life Technologies Corporation, CA, USA).

The samples were subjected to direct automated sequencing using the ABI Prism Genetic Analyzer 3130XL (Life Technologies Corporation, CA, USA). The sequences obtained were compared to those in the National Center for Biotechnology Information database *(NCBI* http://www.ncbi.nlm.nih.gov/*)*. We performed *in silico* analysis of the identified variants using Mutation Taster (available online at http://www.mutationtaster.org).

Comparisons between unrelated patients with POF and controls were performed using contingency tables and χ^2^ analysis for each polymorphism in SigmaStat for Windows v.3.5. *p values*<0.05 were considered statistically significant.

## RESULTS

*NANOS3* mutation screening in 30 unrelated women with POF of unknown etiology identified the rs897790, rs2016163, rs369192674 and rs371590850 variants in 18 patients.

**Figure 1:**
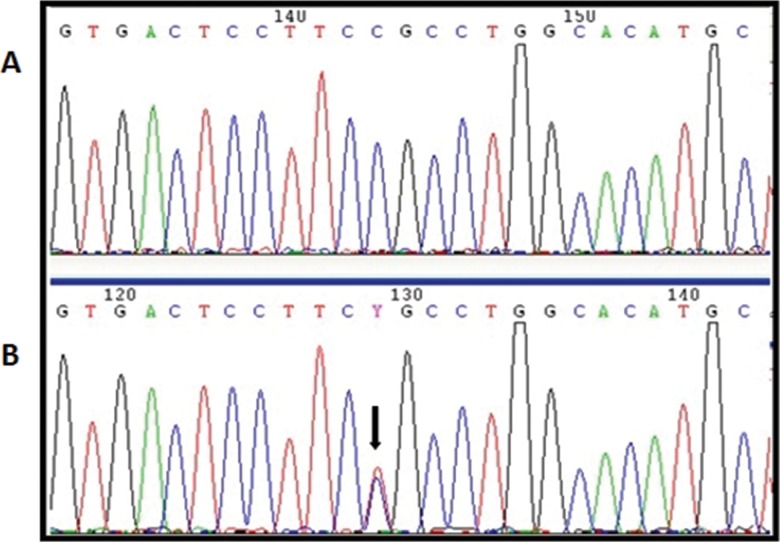
A, Electropherogram of a patient with wild type *NANOS3*. B, Electropherogram of a patient with the heterozygous rs897790 (c.23C>T) variant, indicated by a black arrow.

The rs897790 variant is located in the 5' untranslated region of exon 1, where it caused the replacement of a cytosine with a thymine at position -23 (c.-23C>T); this variant was found in 6 patients in the heterozygous state (Figure 1). In the control group, the rs897790 variant was identified in 59 women, of whom 54 were heterozygous and 5 were homozygous.

**Figure 2:**
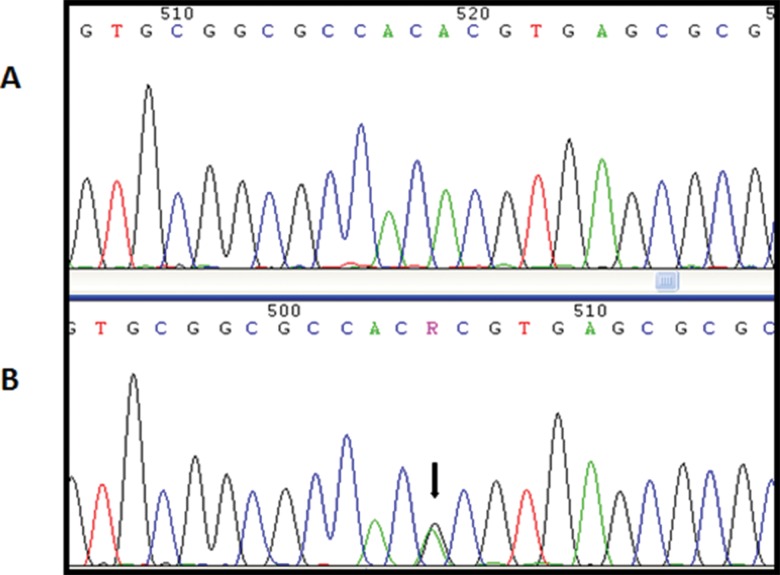
A, Electropherogram of a patient with wild type *NANOS3*. B, Electropherogram of a patient with the heterozygous rs2016163 (c.354A>G) variant, indicated by a black arrow.

The rs2016163 polymorphism is located in exon 1 and causes the substitution of an adenine with a guanine at position 354 (c.354A>G). This substitution does not change the amino acid sequence. Ten of the 30 patients harbored this polymorphism, one in the homozygous state and 9 in the heterozygous state (Figure 2). This variant was present in 70 individuals in the control group, of whom 58 were heterozygous and 12 were homozygous.

Six patients presented with both variants. These previously identified variants are reported in control databases, such as the 1000 Genomes Project, at a frequency of greater than 1%, which classifies them as polymorphisms.

**Figure 3:**
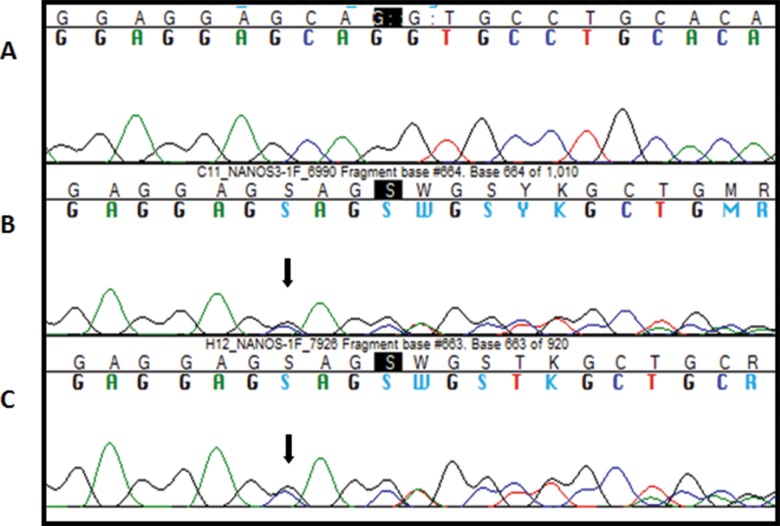
A, Electropherogram of a patient with wild type *NANOS3*. B/C, Electropherogram of a patient heterozygous for the rs369192674 (c.512_514dupGAG) variant. The overlapping sequences of the two alleles, wild type and variant, are shown beginning at the black arrow.

Two patients harbored a GAG duplication at position 512-514 (c.512_514dupGAG) in the transcribed region of exon 1 (rs369192674). This trinucleotide duplication led to the addition of a glycine between positions p.171 and p.172 (p.Gly171dup) (Figure 3). *In silico* analysis of this variant classified it as not deleterious. This variant is described in the NCBI database, although its population frequency is not known. This variant was found in three women in the control group in the heterozygous state.

**Figure 4:**
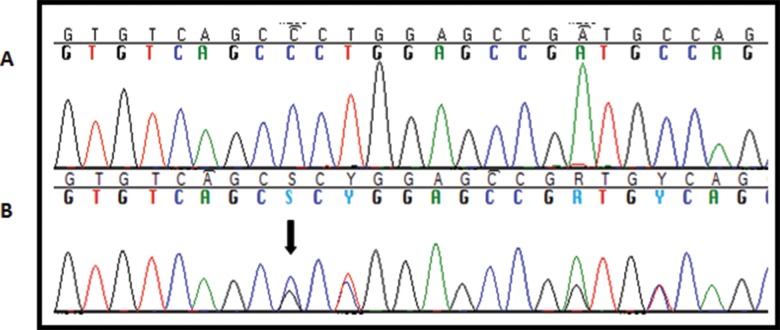
A, Electropherogram of a patient with wild type *NANOS3*. B, Electropherogram of the patient with the heterozygous rs371590850 (c.134_135insGCCGGAGCCGGTGTCAGC) variant. The overlapping sequences of the two alleles, wild type and variant, are shown beginning at the black arrow.

Another patient harbored a heterozygous insertion of 18 nucleotides between positions c.134 and c.135 (c.134_135insGCCGGAGCCGGTGTCAGC) in the transcribed region of exon 1 (rs371590850). This insertion leads to the addition of six amino acids between positions p.45 and p.46 (p.Ala45_Leu46insProGluProValSerAla) (Figure 4). *In silico* analysis classified this variant as not harmful. This variant is described in the NCBI database, although its population frequency is not known. In the control group, this variant was identified as heterozygous in 6 women.

The screening results for the rs897790, rs2016163, rs369192674 and rs371590850 variants in the control and POF groups are summarized in Table 3.

The allele distributions of the rs897790, rs2016163, rs369192674 and rs371590850 variants were not significantly different between the POF patients and the control group (*p*=0.271, *p*=0.787, *p*=0.295 and *p*=0.597, respectively) (Table 3).

We did not identify any deleterious *NANOS3* variants in the studied population.

## DISCUSSION

In the present study, we screened for *NANOS3* mutations in a cohort of women with POF and identified only four previously described variants, which were detected at a similar frequency in the control group.

Although most POF cases are sporadic, 10-15% are familial, corroborating the hypothesis that this disorder has a genetic component. However, despite recent advances in molecular biology, approximately 90% of POF cases are of unknown etiology 1,10. The role of NANOS3 in inhibiting the apoptosis of PGCs and thus contributing to their maintenance makes this protein a natural candidate for the etiology of POF in humans 11. Loss of NANOS3 function may lead to PGC death: *Nanos3* knockout mice do not have PGCs 7. Based on this finding, three groups used Sanger sequencing to screen women with POF for *NANOS3* mutations 8,. Qin et al. studied samples from 80 Chinese and 88 Caucasian women by denaturing high-performance liquid chromatography (DHPLC), followed by Sanger sequencing, and did not identify any causative mutations in *NANOS3* 12. Wu et al. evaluated 100 Chinese patients with POF and reported a heterozygous p.Arg153Trp mutation in *NANOS3* in a 23-year-old patient born from consanguineous parents. Transfection assays in HEK293 cells showed that the half-life of the mutant p.Arg153Trp protein was significantly reduced. Using an animal model, these researchers also showed that the PGC population was directly related to NANOS3 protein levels. Together, these two findings implicate the c.457C>T mutation (p.Arg153Trp) in the etiology of POF in this patient 13.

Finally, a previous study in a Brazilian cohort evaluated 85 patients with primary or secondary ovarian failure and reported the novel homozygous c.358G>A mutation (p.Glu120Lys) in *NANOS3* in two sisters with POF. *In vitro* analysis showed a significant loss of PGC viability, and *in silico* modeling of mutant NANOS3 suggested that this amino acid change might destabilize the protein-RNA interaction. This evidence supports the hypothesis that the c.358G>A mutation, which increases the apoptosis rate of PGCs during the embryonic period, is the etiological factor for POF in these two patients 8.

Considering these three studies involving the index case of 343 families, the *NANOS3* mutation frequency in POF is less than 0.5% 8,.

Our study included 30 additional Brazilian women with POF, but no mutations were found in the *NANOS3* gene. The four variants that were identified in the present study have already been described, but only the rs897790 and rs2016163 variants were previously reported in POF women 8,12,13. The rs2016163 variant was also identified in normal women by Qin et al. 12. No significant difference was observed in the allele frequencies of the rs897790, rs2016163, rs369192674 and rs371590850 variants between POF patients and the control group. Our negative results should be interpreted cautiously due to the small number of patients; POF is a rare disorder. In addition, only the *NANOS3* coding regions and exon boundaries, not the intronic and regulatory regions, were sequenced.

In conclusion, despite the prominent role of *NANOS3* in ovarian development, our findings suggest that *NANOS3* mutations were not associated with POF in the present cohort.

## AUTHOR CONTRIBUTIONS

Sousa BL, Santos MG and Nishi MY performed and analyzed the experimental assays. Brito VN performed the statistical analysis. Sousa BL was responsible for writing the manuscript. Domenice S followed and selected the patients. Mendonca BB designed the project, supervised the study execution and wrote the manuscript.

## Figures and Tables

**Table 1:** Sequences of the primers used to amplify *NANOS3* exons.

Exon		Primer sequence
1	Forward	5′ CTG CTC CTC CCT CTT CAC AC 3′
	Reverse	5′ GTC TTC CCC TAA CCC TTG GA 3′
2	Forward	5′ GTC ACG GGG TCG CTG TCT 3′
	Reverse</emph>	5′ AGT GGG GGC AGT CGT CAT AG 3′

**Table 2:** PCR amplification protocol for the *NANOS3* gene.

Exon	H_2_O (µl)	5X Buffer (µl)	dNTP (mM)	Forward Primer (pmol)	Reverse Primer (pmol)	Taq DNA Polymerase (U)
1	16.3	5	0.2	10	10	0.75
2	16.3	5	0.2	10	10	0.75

dNTP: deoxynucleoside triphosphate; µl: microliters; mM: millimolar; pmol: picomoles; U: units.

**Table 3:** Distribution of *NANOS3* variants in the control and patient groups.

Allelic Variant	POF Group	Control Group	*p*#
rs897790	6*/30**	59*/185**	0.271
rs2016163	10*/30**	70*/185**	0.787
rs369192674	2*/30**	3*/185**	0.295
rs371590850	1*/30**	6*/185**	0.597

POF: Premature Ovarian Failure.

* : number of individuals with the variant;

** : total number of individuals;

# : Chi-square test; *p*<0.05 indicated significance.
